# Characterization and optimization of the haemozoin-like crystal (HLC) assay to determine Hz inhibiting effects of anti-malarial compounds

**DOI:** 10.1186/s12936-015-0913-y

**Published:** 2015-10-12

**Authors:** Carolina Tempera, Ricardo Franco, Carlos Caro, Vânia André, Peter Eaton, Peter Burke, Thomas Hänscheid

**Affiliations:** Faculdade de Medicina de Lisboa, Instituto de Medicina Molecular, Av. Prof. Egas Moniz, 1649-028 Lisbon, Portugal; Departamento de Química, Faculdade de Ciências e Tecnologia, UCIBIO, REQUIMTE, Universidade NOVA de Lisboa, 2829-516 Caparica, Portugal; Centro de Química Estrutural, Instituto Superior Técnico, Universidade de Lisboa, Av. Rovisco Pais, 1049-001 Lisbon, Portugal; , Departamento de Química e Bioquímica, Faculdade de Ciências, REQUIMTE/UCIBIO, Universidade do Porto, 4169-007 Porto, Portugal; STERIS Corporation, 5960 Heisley Road, Mentor, OH 44060 USA; Faculdade de Medicina, Instituto de Microbiologia, Lisbon, Portugal

**Keywords:** Malaria, Haemozoin inhibition, Antimalarial drugs, Simple assay

## Abstract

**Background:**

The haem-haemozoin biocrystallization pathway is an attractive target where several efficacious and safe anti-malarial drugs act. Consequently, in vitro haemozoin (Hz) inhibition assays have been developed to identify novel compounds. However, results may differ between assays and often require complex methods or sophisticated infrastructure. The recently reported growth of haemozoin-like crystals (HLC) appears to be a simple alternative although the endproduct is structurally different to Hz. This study set out to characterize this assay in depth, optimize it, and assess its performance.

**Methods:**

The HLC assay was used as previously described but a range of different growth conditions were examined. Obtained HLCs were investigated and compared to synthetic (sHz) and natural haemozoin (nHz) using scanning electron microscopy, powder X-ray diffraction (PXRD), Fourier Transform Infrared spectroscopy (FTIR) and Raman spectroscopy (RS). Interactions of HLC with quinolines was analysed using RS. Inhibitory effects of currently used anti-malarial drugs under four final growth conditions were established.

**Results:**

HLC growth requires *Mycoplasma* Broth Base, Tween 80, pancreatin, and lysed blood or haemin. HLCs are similar to nHz and sHz in terms of solubility, macroscopic and microscopic appearance although PXRD, FTIR and RS confirm that the haem aggregates of HLCs are structurally different. RS reveals that CQ seems to interact with HLCs in similar ways as with Hz. Inhibition of quinoline drugs ranged from 62.5 µM (chloroquine, amodiaquine, piperaquine) to 500 µM in mefloquine.

**Conclusions:**

The HLC assay provides data on inhibiting properties of compounds. Even if the end-product is not structurally identical to Hz, the inhibitory effects appear consistent with those obtained with sHz assays, as illustrated by the results obtained for quinolines. The assay is simple, inexpensive, robust, reproducible and can be performed under basic laboratory conditions with a simple visual positive/negative read-out.

**Electronic supplementary material:**

The online version of this article (doi:10.1186/s12936-015-0913-y) contains supplementary material, which is available to authorized users.

## Background

Effective treatment is essential for malaria control but resistance to anti-malarial drugs threatens these efforts. This has reached the limelight with reports about the resistance to artemisinins [1, 2], although some controversy exists if this is already true resistance [3, 4]. Still, it underpins the need for new anti-malarial drugs and substantial efforts are made to identify novel compounds [5]. Effective compounds with a novel mode of action (MOA) have been discovered and some are currently undergoing clinical trials [6]. New drugs also have to prove that they are sufficiently safe before widespread use, a crucial issue highlighted by the recent description of delayed haemolysis after artemisinin treatment [7].

Discovery of new compounds which target known pathways, where existing drugs have been shown to be efficacious and save, is another option. The prime example is the parasite specific haem-haemozoin bio-crystallization pathway where many of the anti-malarial drugs seem to act, including quinine, chloroquine, mefloquine, amodiaquine, lumefantrine, halofantrine, and piperaquine [8, 9]. This pathway appears immutable and resistance is caused by removal of the compound from its site of action, as in the case of chloroquine (CQ) [10]. Interestingly, this contrasts with drugs which have a different MOA, like atovaquone or sulfadoxine/pyrimethamine, where mutations cause alterations in the target [11, 12].

The haemozoin (Hz) formation pathway has been extensively studied as summarized in several superb reviews [10, 13–16] including the putative, yet controversial role of artemisinins [17, 18]. However, no complete consensus exists about the Hz pathway. Several common themes have emerged [13, 14] including: Hz biocrystallization is autocatalytic; involvement of lipids, growth at the lipid aqueous interface, orientated nucleation at the digestive vacuole (DV) membrane and a presumed role for a haemozoin detoxification protein (HDP). Furthermore, all these proposed mechanisms have to be reconciled with other aspects. For example, several organisms (*Schistosoma, Rhodnius, Haemoproteus*) produce Hz, apparently without a digestive vacuole, and the resulting Hz looks morphologically different, even if it has an identical crystal structure [19, 20]. Another aspect is the fact that Hz does not grow motionless in the DV, but apparently shows a rapid non-Brownian movement [21].

Synthetic haemozoin (sHz), or β-haematin, is structurally identical to haemozoin [22]. Based on the protocol to synthesize it, it tends to form larger units that can range from 50 nm up to 20 µm [13, 23]. Several assays based on sHz production exist to assess the haemozoin inhibition of compounds [24–30]. However, standardization has been difficult and results, including inhibitory concentrations, have shown variations between assays (see Additional file 1) [24, 25, 27, 31–33]. Assay conditions varied including different reagents, pH and incubation times, or different types of initiators [24–30]. Further issues are the use of rather toxic reagents like pyridine [25] or a rather complex readout: the produced haemozoin is washed, transformed back into haem and then read spectrophotometrically [26, 29, 34]. Although, a recent high throughput assay has produced interesting results, it included expensive instruments and robotics [34]. This may be justified when screening thousands of compounds, yet, often research efforts may involve the verification of haemozoin inhibiting effects in fewer compounds [35], or in conditions where these high throughput methods are not easily available [36].

As a possible alternative a simple and low-cost assay based on the formation of so-called Hz-like crystals (HLC) has been reported [37]. Although HLC seem to be structurally different to Hz, a range of reported inhibitory effects of antimalarial compounds appears to be consistent with those obtained with sHz inhibition assays [37].

In the 1980s work on a “replicating agent”, termed “Ileal Fluid Dependent Organism” (IFDO) was described [38]. This method was re-investigated in the quest to find a novel and simple replacement assay for the currently used complex, time intensive and expensive in vivo and in vitro assays to evaluate the therapeutic and decontamination efficacy against transmissible misfolded proteins (prion diseases) [39]. Interestingly, quinoline derivatives and cyclins presented anti-prion activity in vitro [40–42].

Given the structural difference of HLC and Hz, yet the apparently consistent inhibitory results for some quinolone compounds, the objective of this follow up study was to characterize the assay in more detail, as well as optimizing the assay in terms of establishing the essential components, decrease turn-around time, and test the newly established protocol to determine HLC-inhibitory effects of anti-malarial drugs.

## Methods

### Synthetic haemozoin (sHz)

Synthetic haemozoin (sHz) was obtained as previously described [43], with some modifications. Briefly, 475 mg of haemin chloride (Sigma Aldrich, St. Louis, MO, USA) was dissolved in 100 ml of 0.1 N NaOH and haem was precipitated by slowly adding 35 ml of glacial acetic acid. After overnight incubation at 80 °C, non-crystalline haem was removed by washing three times with 1 vol. of 100 mM sodium bicarbonate (pH 9.1) during 3 h. The obtained sHz was further washed in ultrapure water obtained with a Milli-Q purification system (Millipore, Madrid, Spain) and stored at 4 °C.

### Haemozoin (Hz) from *Plasmodium falciparum*

*Plasmodium falciparum* haemozoin was harvested after saponin lysis of *P. falciparum* cultures (3D7 strain) as previously described by Coban et al. [44]. In short, after extensive washes in phosphate buffered saline (PBS) (Gibco, Grand Island, NY), the pellet was sonicated for 5 min, followed by further extensive washes with 2 % sodium dodecyl sulfate (SDS) and then incubated overnight with 2 mg/ml Proteinase K. After being washed with 2 % SDS again, the pellet was incubated for 3 h in 6 M urea and then washed with 2 % SDS and ultrapure water.

### Baseline haemozoin-like crystal (HLC) growth medium

The culture medium was adapted from the original protocol for growing Ileal Fluid Dependent Organisms (IFDO) [38], as described by Thomas et al. [37] for HLC growth. Briefly, 35.5 g of *Mycoplasma* Broth Base CM403 (Oxoid Ltd., Basingstoke, Hampshire, England) was dissolved in 950 mL of distilled water with 2 mL/L of Tween 80 (Fluka, Buchs, Switzerland) and then autoclaved. Red blood cells from consenting healthy human donors were washed three times in 1× PBS (Gibco, Grand Island, NY, USA), followed by lysis using sterile distilled water and then submitted to three freeze/thaw cycles, followed by five minutes of sonication in an ultrasound bath. After centrifugation at 3200*g* for 15 min the supernatant was obtained and kept at −20 °C. Pancreatin (4X UPS) (Sigma Aldrich, St. Louis, MO, USA) was prepared as 10 % solution in 1× PBS, followed by filtration with a 0.22 µm syringe filter (Millipore, Madrid, Spain). To the cooled broth, 30 mL of the lysed blood, 20 mL of the 10 % pancreatin and 1.33 mL horse serum (Gibco, Grand Island, NY, USA) were added. To initiate the growth, the medium was “seeded” with previously obtained HLC with 0.1 µM haem equivalents, distributed in plates, and incubated at 37 °C, 5 % CO_2_ for up to 5–7 days. HLCs for seeding were obtained by washing the final crystalline product three times with 1 vol. of 100 mM sodium bicarbonate (pH 9.1) during 3 h, followed by three times using ultrapure water. Measurement of haem-equivalents, after solubilization in 20 mM NaOH for 1 h, was done using the QuantiChrom™ Heme Assay Kit (Bioassay Systems, Hayward CA, USA).

**Fig. 1:**
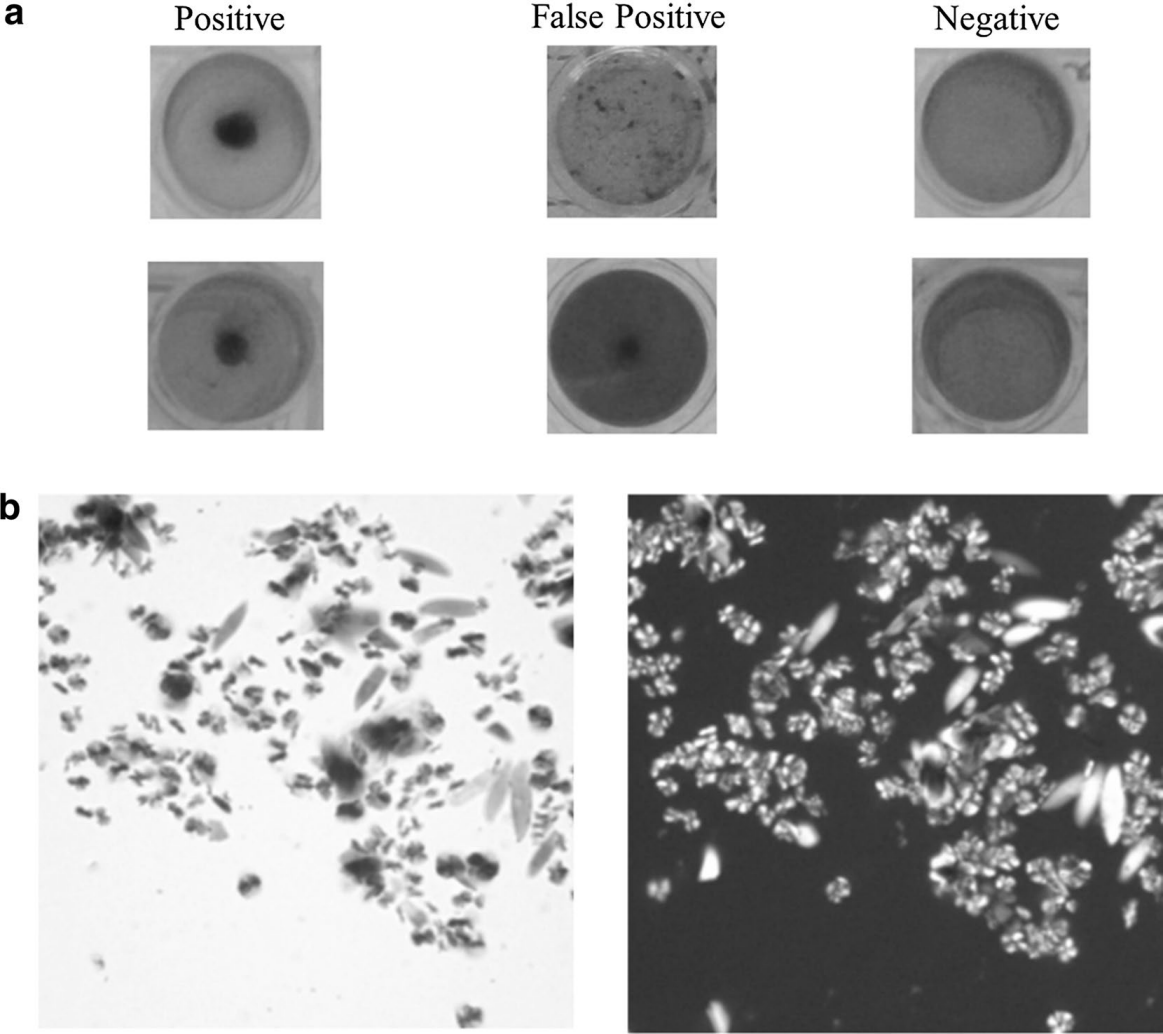
**a** Medium after incubation. Images on the *left* represent the expected growth, a *black* sediment in a clear medium (positive). *Central images* show a false positive growth where the sediment does either not agglomerate or is hardly discernable in a dark medium of a *color* similar to the non-seeded control at the beginning of the incubation. Images on the *right* show the absence of growth (negative). **b** Light microscopic image of HLC (*left*) and under polarized light (*right*) at 1000x amplification, showing typical depolarization, absent in atypical deposits.

The assay readout was visual inspection of the sediment and a well with visible black pigment was considered positive (Fig. 1A). Black sediment in wells was observed microscopically (Leica DM2500) for size and form of crystals, only were considered as HLC if they depolarized light (Fig. 1B).

### Investigation of different growth conditions and essential components

A variety of different growth conditions were tested (see Additional file 2, for complete list and results), including: different pH, incubation at different temperatures, atmospheres, different sources of blood, replacing the lysed blood by haemin dissolved in DMSO at various concentrations 10, 5 and 2.5, different pancreatin concentration (2.5 and 10 %), various amounts of Tween 80, or using synthetic haemozoin as seeding instead of IFDO/HLCs (see Additional file 2, for complete list). The growth was monitored daily by visual inspection until 7 days of incubation (see Additional file 3, for images of a time course), to establish the optimal period necessary for the assay.

### Characterization of grown haemozoin-like crystals

Resulting HLCs were investigated for their stability using diverse acids (sulphuric acid, acetic acid), solvents (DMSO) and alcohols (Ethanol, Methanol). HLC and some of the resulting growth, obtained under different conditions, was analysed by scanning electron microscopy and X-ray diffraction and compared with sHz and nHz. Raman spectroscopy was also performed to analyse the HLC, alongside with the sHz and nHz, as well as their interaction with chloroquine (Sigma-Aldrich, St. Louis, Mo, USA).

### Scanning electron microscopy: SEM

Samples were centrifuged at 9000 rpm for 120 s and the pellet re-suspended in 250 µL methanol of which 40 µL were deposited on a carbon tape substrate. Crystals were not coated prior to scanning. Samples were imaged in a FEI Quanta 400 FEG ESEM/EDAX Genesis X4 M at high vacuum, using 15 kV accelerating voltage, and approximately 10 mm working distance. Images were collected in secondary electron (SE) mode.

### Powder X-Ray diffraction

Crystalline powders were obtained through suspension in methanol. Data was collected in a D8 Advance Bruker AXS θ–2θ diffractometer, with a copper radiation source (Cu Kα, λ = 1.5406 Å) and a secondary monochromator, operated at 40 kV and 40 mA, within the 5–35° range in 2–θ.

### FTIR experiments

For FTIR experiments, 10 µL of a concentrated solution of each sample was deposited on a glass slide and dried at room temperature. This powder was scrapped and compacted into a Thermo diamond anvil compression cell. Infrared analyses were carried out with a Nicolet Nexus spectrophotometer coupled to a Continuμm microscope (15× Objective) with a MCT-A detector cooled by liquid nitrogen. Micro-samples were collected and spectra were obtained in transmission mode, 4000–650 cm^−1^, with a resolution of 4 cm^−1^ and 128 scans. Spectra are shown here as acquired, without corrections or any further manipulation.

### Raman experiments

Raman spectra were acquired with a Raman setup (HR LabRam, Horiba Jobin–Yvon) equipped with an Olympus IX71 microscope, a video camera and liquid nitrogen cooled CCD detector. An Olympus 50X objective focused the laser light on the samples. As excitation wavelength, the 633 nm line of a HeNe laser was used. Calibration of the wavenumber axis was performed with a silicon sample. All measurements were collected from randomly selected spots of the samples deposited on a glass slit. Each spectrum was the accumulation of five scans of 25 s laser exposure each.

For Raman experiments, 10 µL of a concentrated solution of each sample was deposited on a glass slide and dried at room temperature. A concentrated solution of each sample (10 µL) was incubated with a 20 mM chloroquine (Sigma-Aldrich, St. Louis, Mo, USA) aqueous solution to study interactions. After 30 min of incubation, 10 µL of mixture was deposited over a glass slide, air-dried and analysed. A more detailed description of the Raman analysis can be found in Additional file 4.

### Testing compounds with the HLC inhibition assay

All compounds were purchased from Sigma-Aldrich (St. Louis, Mo, USA) except for mefloquine (Roche, Mannheim, Germany) and piperaquine (Sigma-tau, Rome, Italy). Stock solution of chloroquine, amodiaquine, piperaquine and tetracycline were dissolved in water; quinine in DMSO; mefloquine, halofantrine, artemisinin, artesunate in methanol; and rolytetracycline and doxycycline in ethanol 70 % and pyrimethamine in absolute ethanol. Based on the investigation of growth conditions, these compounds were tested with: (1) the baseline medium, (2) blood extract substituted with haemin at 5 mM in DMSO, (3) pancreatin 4× USP (Sigma-Aldrich) at 10 % and (4) 2.5 %. A range of doubling concentrations from 15.6 to 1000 µM was tested under these four conditions. After preparing the seeded mediums with the compounds serial dilutions, were incubated for 5–7 days at 37 °C in 5 % CO_2_. The plates were observed daily. The results from the 48 h incubation time points were used to establish inhibition.

## Results

### Growth of haemozoin-like crystals

From the originally described medium to grow IFDO, only four ingredients are necessary for growth: *Mycoplasma* Broth Base, Tween 80, lysed blood and pancreatin (Table 1). Lysed blood can be from diverse species, including horse or sheep, or it can be substituted with haemin. Interestingly, without *Mycoplasma* Broth, some black deposit forms but it does not appear to be similar to HLC, also indicated by its lack of depolarization. Lysed blood or haemin, with 10 or 2.5 % pancreatin appear to give best results (Table 1) and were the conditions used for the drug inhibition studies.

**Table 1:** Growth effect of different ingredients of the HLC assay

*Mycoplasma* Broth	Tween 80	Lysed blood	Pancreatin (4× USP)	Serum	Growth results
Yes	Yes	Yes	Yes	Yes	Baseline condition
Yes	Yes	Yes (s/h)	Yes	Yes	Like baseline
No	Yes	Yes	Yes	Yes	Little growth, no depolarization
Yes	No	Yes	Yes	Yes	No growth
Yes	Yes	No	Yes	Yes	No growth
Yes	Yes	Yes	No	Yes	No growth
Yes	Yes	Yes	Yes	No	Like baseline
Yes	Yes	Hem	Yes	No	Like baseline
Yes	Yes	Hem	Pan 2.5 %	No	Slightly better than baseline
Yes	Yes	Yes	Pan 2.5 %	No	Slightly better than baseline

Baseline conditions as described for the IFDO assay (see text for details): *Mycoplasma* Broth, Tween 80, lysed blood, pancreatin 10 % (4× USP), horse serum, seeded with 0.1 µM of HLCs, incubated for 5–7 days. Results of 3–5 independent experiments

*(s/h)* replacement of Human blood with sheep (s) or horse (h) blood, *Haem* lysed blood substituted with haemin (5 mM) in DMSO, *Pan 2.5* *%* pancreatin 10 % substituted with pancreatin 2.5 %

Visible growth of the HLC becomes noticeable at around 24 h if the medium is seeded with HLC. Around 3/4 days, growth occurs spontaneously in the medium in the absence of seeding. After 4 days, there appears to be no change in visible growth until the end of the incubation period (see Additional file 3, for time course of incubated plates).

An extensive set of conditions was investigated for its growth influencing effect, however, none of these improved the growth of HLC, while many produced inferior results (see Additional file 2, for detailed results).

### Characterization of HLC and comparison with sHz and nHz

HLC is a black pigment, macroscopically indistinguishable from sHz or nHz. Microscopically, it consists of birefringent crystalline like material which depolarizes light (Fig. 1b). HLC does not dissolve in DMSO, methanol, ethanol, nor under acidic conditions. As expected, and similar to nHz or sHz it dissolves readily in a strongly alkaline solution, at a pH > 10.

### Scanning electron microscopy imaging

**Fig. 2:**
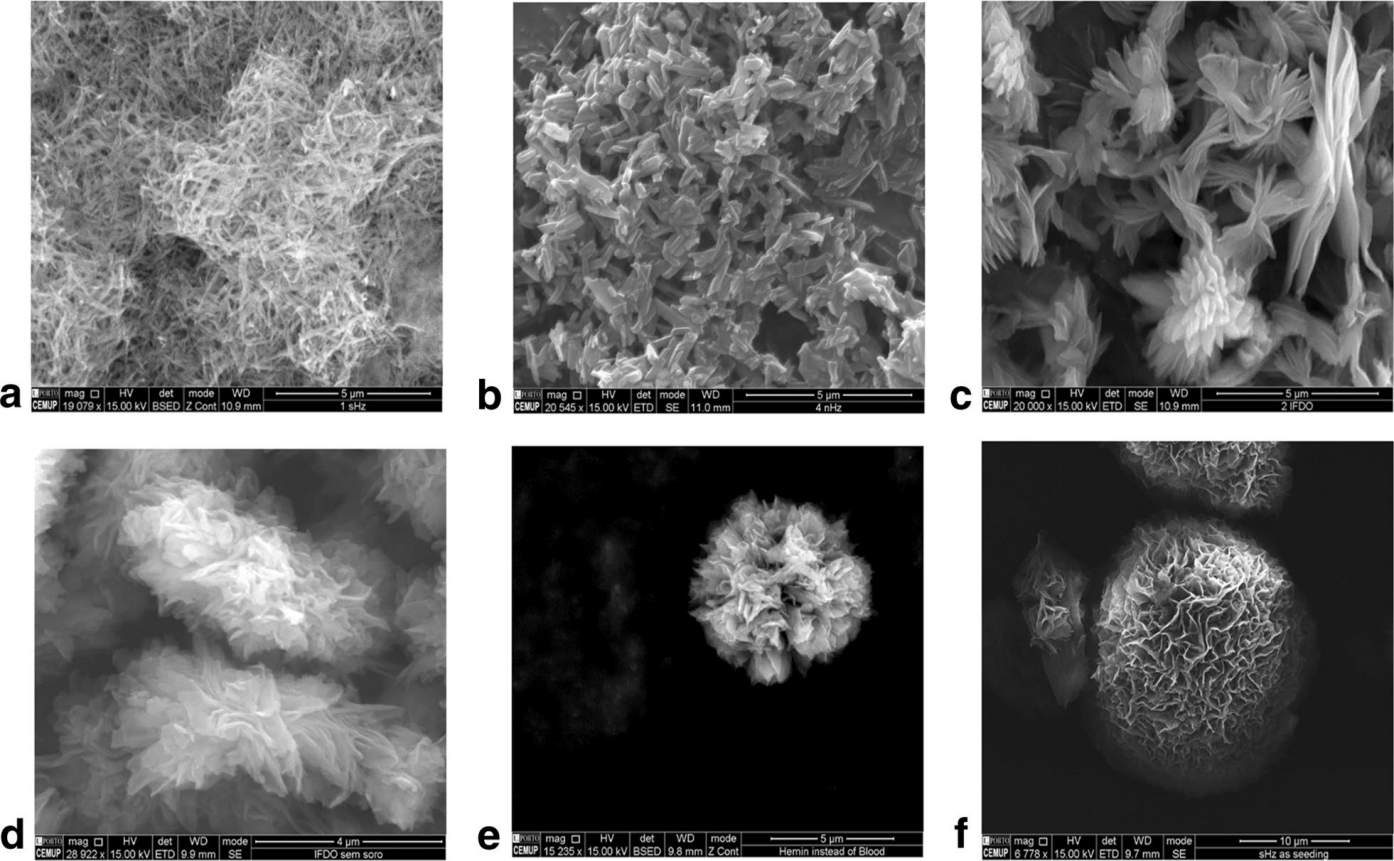
SEM images of haemozoin and different types of haemozoin-like crystals. *Top row* **a** Synthetic haemozoin, **b** natural haemozoin purified from *P. falciparum* culture (3D7), and **c** haemozoin-like crystals. *Bottom row* **d** HLC growth without serum, **e** HLC growth with haemin instead of lysed blood, and **f** HLC with sHz instead of HLC as initial seeding. See text for description of methods

The sHz sample consisted of mats of fine needle-like crystals where individual crystals appear flat and slightly wider in the middle than at the edges, 700–1000 nm long and 60–90 nm wide (Fig. 2a). The nHz looked similar but the needles were shorter and more square in profile, around 350–1000 nm long and 150–250 nm wide (Fig. 2b). The HLCs showed a different arrangement, where crystals form flat needles of up to 1500 nm in length. They appear to be arranged around a central point, rather like “petals of flower” with a total diameter of 3–4 µm (Fig. 2c).

HLC grown under different conditions are shown in Fig. 2, bottom row. Without serum, “flower” like structures are also visible, 4–8 µm across (Fig. 2d), also seen when grown with haemin, although the “petals” appear more organized, forming a globular structure (Fig. 2e). Using sHz as seeding also produced similar agglomerates (Fig. 2f).

### Powder X-Ray diffraction of sHz, nHz and HLC

**Fig. 3:**
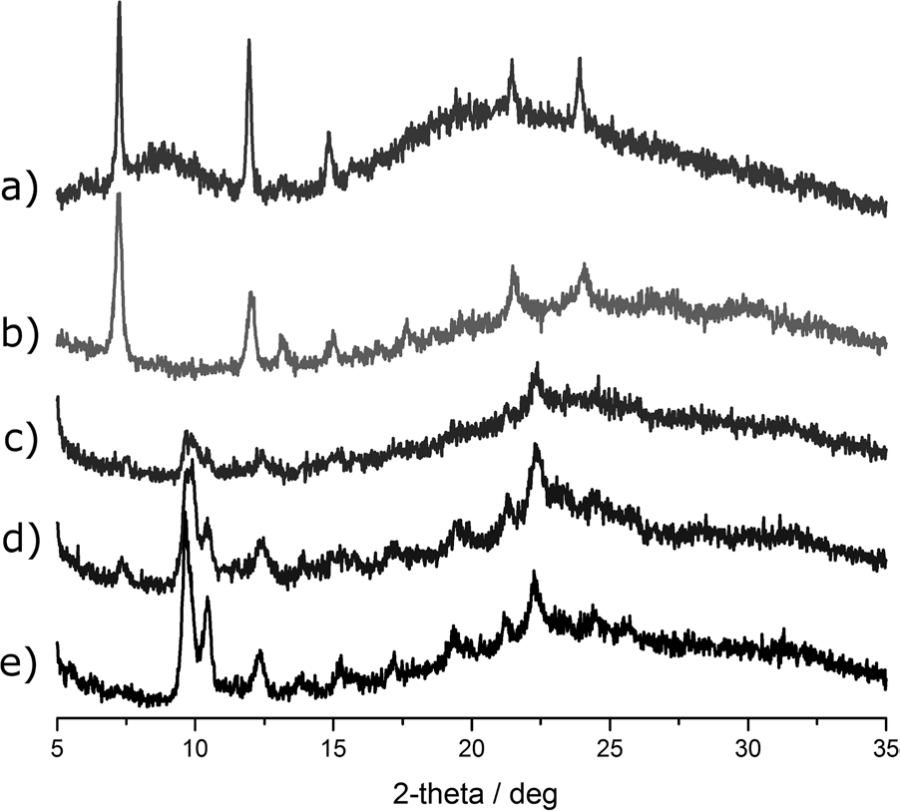
X-ray diffraction of sHz, nHz and HLC. Result for *a* natural Hz, *b* synthetic Hz, *c* spontaneous growth of haemozoin-like crystals (no seeding), *d*, *e* two independent productions of haemozoin-like-crystals. As expected nHz and sHz show identical results which are different from HLC, confirming the different internal structure

Despite some amorphous content detected in the background of the diffractograms, it is apparent that sHz and nHz are identical (Fig. 3) and thus have the same crystalline structure, yet they are different from all HLC samples. All HLC samples, including those where (1) sHz was used as seeding, (2) spontaneous growth of HLC occurred, and (3) HLC produced with haemin instead of lysed blood, have identical patterns (Fig. 3). None of these HLC samples appears to be mixed with sHz phases, as sHz specific peaks are not found in the powder diffraction patterns.

### FTIR spectroscopy of haemin, sHz, and HLC

**Fig. 4:**
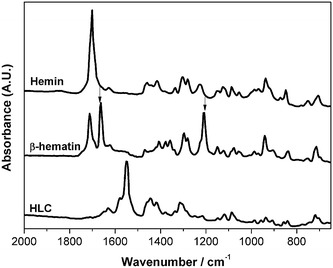
FTIR spectra of haemin, sHz and HLC. Fourier Transform Infrared spectroscopy: Haemin (*a*), synthetic Hz also called β–Haematin (*b*); and Hemozoin-Like Crystals (HLC) (*c*). *Vertical arrows* indicate typical bands associated with sHz and nHz, which are practically absent in the spectrum of HLC

Figure 4 presents FTIR spectra of haemin (Fe(III)-protoporphyrin IX), sHz (β–haematin), and HLC. Spectra of haemin and sHz are similar to those previously reported. Namely, the sHz spectrum clearly show bands at 1664 and 1209 cm^−1^ assigned to the carbonyl stretching mode and the C-O stretching vibration of the propionate linkage, respectively, and that were also observed in nHz [45]. The FTIR spectrum of HLC presents only very weak shoulders at these energy values. Nevertheless, all three spectra exhibit a common band at 1625 cm^−1^, which appears as a strong line in the Raman spectrum.

### Raman spectroscopy of haemin, sHz, nHz and HLC and interaction with chloroquine

**Fig. 5:**
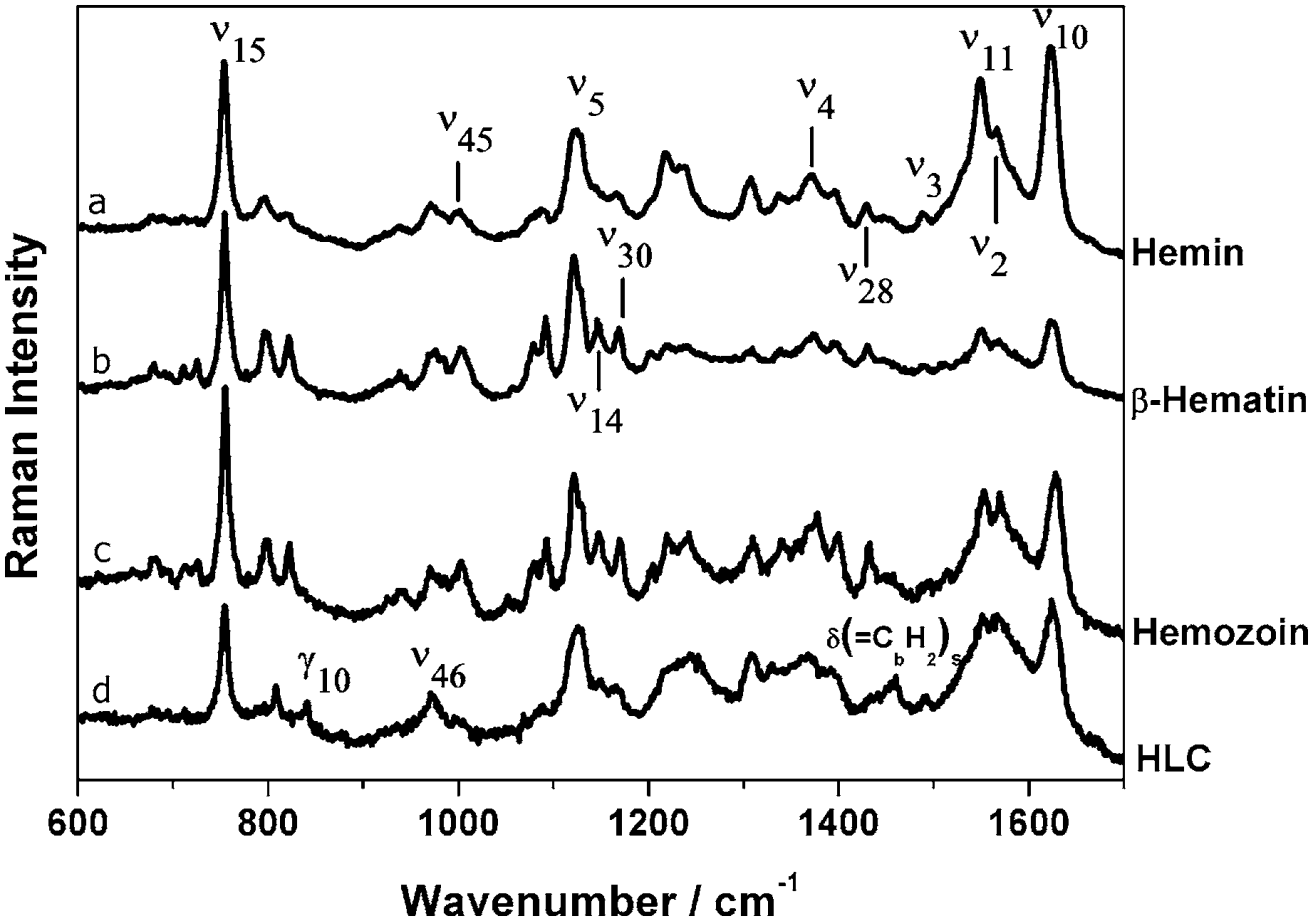
Raman spectra of haemin, sHz, nHz and HLC. Haemin (*a*), synthetic haemozoin also called β–Haematin (*b*), native haemozoin from *P. falciparum* (*c*); and Haemozoin-like crystals (HLC) (*d*). Spectra were obtained using 632.8 nm laser excitation. Identical *lines* of sHz and nHz, while HLC shows a different pattern. For a detailed description of line assignments see text and Additional file 5

Raman spectroscopy provides useful structural information based on molecular vibrational states and Fig. 5 presents Raman spectra of haemin (Fe(III)-protoporphyrin IX), sHz (β–Haematin), nHz, and HLC. The more ordered structure of haemin in nHz and sHz is reflected in sharper and better resolved Raman lines, in relation to the broader and less resolved Raman lines observed in HLC and free haemin (Fig. 5). Detailed analysis of the Raman results, including line assignments, symmetry terms and local coordinates can be found in Additional file 5.

**Fig. 6:**
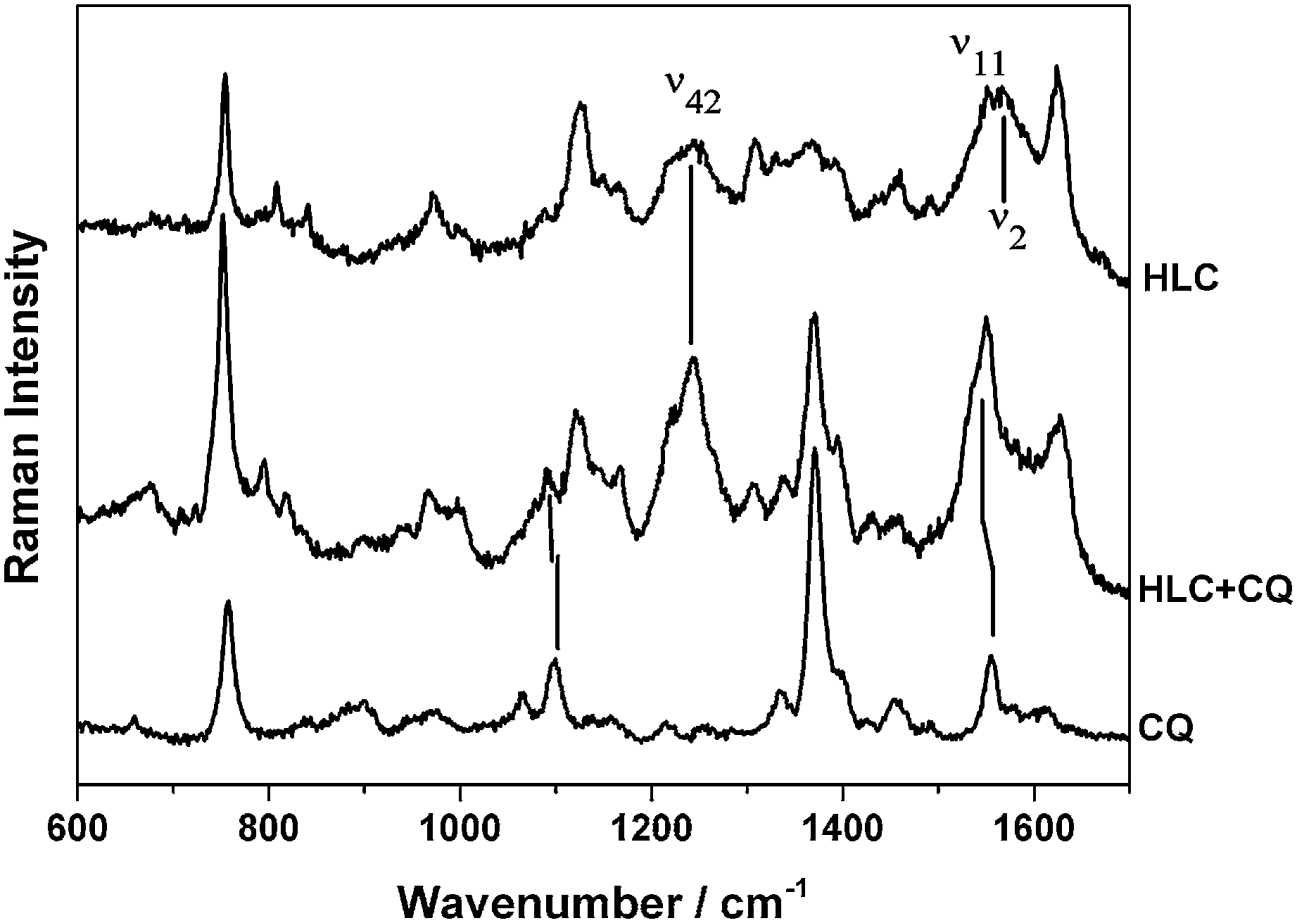
Raman spectra of HLC interactions with chloroquine. Haemozoin-like crystals (*a*), haemozoin-like crystals incubated with chloroquine (*b*), and chloroquine alone (*c*). Spectra were obtained using 632.8 nm laser excitation. *Vertical lines* indicate Raman line shifts. For a detailed description of line assignments see text and Additional file 5

Chloroquine (CQ) interactions with HLC are shown in Fig. 6. Some line shifts and intensity changes can be observed in the Raman spectrum of the HLC/CQ mixture that are diagnostic of interaction between haemin in HLC and CQ. The most obvious is the much increased intensity of the Raman line at 1242 cm^−1^, assigned to ν_42_, a vinyl-associated vibration with an E*u* symmetry term (see Additional file 5). This line intensity increase occurs also for CQ-incubated haemin and sHz, but not for nHz (see Additional file 6). Comparing the spectrum of CQ alone with the HLC/CQ mixture (Fig. 6, spectra C and B), some line shifts are also observed. Namely, the line at 1556 cm^−1^ in CQ red-shifts and appears as a shoulder in the low-frequency side of the ν_11_ line of HLC. The same can be observed in the Raman spectra for CQ incubated with haemin, sHz or nHz (see Additional file 6). On the other hand, the line at 1086 cm^−1^ in CQ red-shifts as much as 10 cm^−1^. Such shifts seem to indicate that the interaction between haemin molecules in HLC and CQ occurs mainly via a pyrrole-quinoline ring π-π interaction.

### Performance of the HLC assay to assess inhibition by antimalarial compounds

Based on the optimization experiments (Additional file 2), four medium conditions were used to test the inhibitory effect of anti-malarial drugs: lysed blood (Table 2, top half) or with haemin 5 mM (Table 2, bottom half). Both conditions were tested using pancreatin at 2.5 % (Table 2, left part in columns) and 10 % (Table 2, right part in columns). The 4-amino-quinolines and amino-alcohols inhibited growth, with chloroquine and amodiaquine being the most potent at 62.5 µM. Interestingly, artemisinin and artesunate also showed inhibitory activity. Tetracyclines, inhibited growth weakly, albeit only at the highest concentrations. As expected, gentamycin and pyrimethamine showed no inhibitory action, and gentamycin was used as negative control in all experiments.

**Table 2:** Inhibitory effects assessed with the HLC assay

μM	15.6		31.25		62.5		125		250		500		1000	
	P2.5	P10	P2.5	P10	P2.5	P10	P2.5	P10	P2.5	P10	P2.5	P10	P2.5	P10
Blood														
Chloroquine	+	+	+	+	–	–	–	–	–	–	–	–	–	–
Amodiaquine	+	+	+	+	–	–	–	–	–	–	–	–	–	–
Quinine	+	+	+	+	–	+	–	–	–	–	–	–	–	–
Piperaquine	+	+	+	+	–	–	–	–	–	–	–	–	–	–
Halofantrine	+	+	+	+	+	+	+	–	–	–	–	–	–	–
Mefloquine	+	+	+	+	+	+	+	+	–	+	–	–	–	–
Artemisinin	+	+	+	+	+	+	+	+	+	+	–	–	–	–
Artesunate	+	+	+	+	+	+	+	–	–	–	–	–	–	–
Doxycycline	+	+	+	+	+	+	+	+	+	+	–	+	–	–
Rolitetracycline	+	+	+	+	+	+	+	+	+	–	–	–	–	–
Tetracycline	+	+	+	+	+	+	+	+	+	–	+	–	+	–
Pyrimethamine	+	+	+	+	+	+	+	+	+	+	+	+	+	+
Gentamicin	+	+	+	+	+	+	+	–	+	–	+	–	+	–
Haemin														
Chloroquine	+	+	+	+	–	–	–	–	–	–	–	–	–	–
Amodiaquine	+	+	+	+	–	–	–	–	–	–	–	–	–	–
Quinine	+	+	+	+	+	+	–	–	–	–	–	–	–	–
Piperaquine	+	+	+	+	+	–	–	–	–	–	–	–	–	–
Halofantrine	+	+	+	+	+	+	+	+	–	–	–	–	–	–
Mefloquine	+	+	+	+	+	+	+	+	–	+	–	–	–	–
Artemisinin	+	+	+	+	+	+	+	–	–	–	–	–	–	–
Artesunate	+	+	+	+	+	–	–	–	–	–	–	–	–	–
Doxycycline	+	+	+	+	+	+	+	+	+	+	–	+	–	–
Rolitetracycline	+	+	+	+	+	+	+	+	+	–	+	–	+	–
Tetracycline	+	+	+	+	+	+	+	+	+	+	–	–	–	–
Pyrimethamine	+	+	+	+	+	+	+	+	+	+	+	+	+	+
Gentamicin	+	+	+	+	+	–	+	–	+	–	+	–	+	–

Inhibitory effects of compounds assessed using the standard medium (see text for details). Doubling concentrations ranged from 15.6 to 1000 μM. Left columns (P2.5) contained pancreatin 4× USP at 2.5 % and right columns (P10) contained pancreatin 4× USP at 10 %. Top half to the table shows results using lysed blood and bottom half using haemin. Growth (+) and no growth (−)

When the lysed blood is replaced by haemin the inhibition appeared to be more pronounced in some instances, although maximally by one concentration step (compare top half with bottom half, Table 2) and the results appeared slightly more reproducible (see Additional file 7). The higher pancreatin concentration seemed to increase the inhibitory effect of some drugs by one concentration (Table 2, left–right part of columns). This was observed, for example, in the case of artesunate as well as for rolitetracycline and tetracycline, although here only in the presence of lysed blood. The resulting inhibitory concentrations were of 62.5 µM for chloroquine, 62.5 µM for amodiaquine, 125 µM for quinine, 500 µM for mefloquine, 250 µM for artemisinin, and 125 µM for artesunate (at 2.5 % pancreatin).

## Discussion

In the 1980s, a medium was devised to grow so-called ileal-fluid-dependent-organisms (IFDO) in an attempt to detect an agent associated with Crohn’s Disease [38]. This procedure and the medium were recently re-investigated in an attempt to produce a simple alternative to screen compounds for their prion-inhibiting activity (see supplementary data in [37]). The original liquid medium was formulated empirically and consisted of *Mycoplasma* Broth Base, Tween 80, lysed horse blood, pancreatin and horse serum [38]. Later, to start the growth, already grown IFDO was added to “seed” the medium [37]. Rather than a microorganism, subsequently it was found that IFDO consists of some kind of haem containing crystal (haemozoin-like crystal) and that some anti-malarial drugs inhibited the growth [37].

The current anti-malarial drug evaluation study shows the essential components of the original medium are *Mycoplasma* Broth Base, Tween 80, lysed blood which can be substituted by haemin, and pancreatin (see Additional file 2). Interestingly, without *Mycoplasma* broth some black deposit forms, which is different from HLC because growth is more disperse and importantly, it does not depolarize, contrary to HLC (Fig. 1b). A wide range of conditions was tested to establish if they would improve the growth and thus the assay, but no apparent advantage was observed (see Additional file 2). When the seeding with HLC was substituted by sHz, the end result was also HLC (Figs. 2, 3). In fact, after 3/4 days of incubation, “spontaneous” growth of HLC occurred consistently in non-seeded medium. All HLCs showed identical X-ray diffraction pattern (Fig. 3) suggesting the resulting structure of the crystal is not determined by the initial seeding material, which only accelerates the growth. Powder X-ray diffraction patterns for sHz, nHz and HLC present some contribution from an amorphous phase, which is likely due to the presence of lipids or less crystalline amorphous Hz pigments that may present in the bulk. Previous reports have already shown that the background of powder X-ray diffraction measurements of the malaria pigment in red blood cells infected with *P. falciparum* display a similar effect [46, 47].

The fundamental component is haem and was included in the form of lysed blood. Neither anti-coagulated human blood from different volunteers, nor the use of defibrinated sheep or horse blood influenced the results (Additional file 2). The lysed blood could also be substituted by bovine haemin, giving comparable results (Table 2). This appears to exclude the possibility that rests of erythrocyte membranes in the lysed blood play any important role, an interesting observation given that several reports implicate membranous structures in the growth of Hz, both in vitro [48] and in vivo [49, 50]. Therefore, the assay appears versatile enough to work with many sources of haem. Due to some possible misperception in previous work [37, 38] concerning the concentration of the used pancreatin (1× or 4× US Pharmacopeial Convention or USP [51]), two final concentrations, 2,5 and 10 %, were used and both produced good growth (Table 2).

Pancreatin is a poorly defined extract of porcine origin containing several enzymes which can be classified in four groups: nucleases (e.g. ribonucleases), glycosidases (e.g. amylase, glucosidase), peptide hydrolase/peptidases (e.g. trypsin, chymotrypsin) and lipolytic enzymes like lipases [52]. Similarly, *Mycoplasma* broth base contains many non-chemically defined substances, with each litter containing: 10 g bacterial peptone (derived from enzyme treated milk or meat), 10 g LAB-LEMCO powder (meat extract), 5 g NaCl, and 0.5 g mineral supplement [53]. Most of this appears to be proteins and it is difficult to establish which or why any of these components are necessary when compared to those necessary for in vitro sHz growth. These include diverse lipids [29, 54–57], membranes or extracts containing membranes [48, 49] and/or diverse detergents [57–59]. In nHz formation a role for a putative haemozoin detoxification protein (HDP) has been described [60]. However, it appears unlikely that this or a similar protein would be present in the *Mycoplasma* broth or pancreatin. Perhaps, a role for the lipase from the pancreatin could be conjectured. Tween 80 (polysorbate 80) is polyethoxylated sorbitan and oleic acid [61] and perhaps, lipase liberates the oleic acid which has been shown to induce growth of sHz [57]. Notwithstanding, Tween 80 itself has been shown to aid sHz growth through its detergent properties [61], which might also be relevant in the HLC growth medium.

HLC is macroscopically and microscopically very similar to Hz, including its birefringence and the resistance to many chemical agents as described for Hz. However, the crystal structure of sHz and that of diverse natural haemozoins from different species appears to be the same [19, 20, 22]. Contrary to this, X-ray diffraction, FITR and Raman spectroscopy strongly suggests that HLCs present a different organization of the haemin monomers relative to sHz and nHz (Figs. 3, 4, 5). SEM images of HLC show a “flower-like” crystal structure, somewhat larger than agglomerations of sHz or nHz (Fig. 2). However, while sHz and nHz are often described as typically needle or brick-shaped [13], comparing Hz from different *Plasmodium* species [23] or organisms like *Schistosoma*, *Rhodnius* or *Haemoproteus* [19, 20] shows a substantial variability.

Perhaps, the most remarkable aspect of HLC is rather how similar it is to Hz in some aspects, despite the obvious differences in molecular structure. Although synthetic and natural haemozoins appear to be structurally identical, no consensus exists yet regarding the exact mechanism that leads to Hz formation in vivo [13]. Moreover, any hypothesis has to reconcile the formation of Hz in all species, despite different morphological and physiological conditions [19, 20]. For example, *Plasmodium* produces Hz in a DV, while *Schistosoma* produces it in the gut. Perhaps even more important, the components and mechanism described to produce synthetic Hz in vitro may not reflect at all the true process in vivo [21]. This is nicely illustrated by the recently described vigorous movement of Hz inside the DV [21] as this has to be reconciled with ideas of oriented growth on surfaces, such as membranes or lipid particles [50, 56] which seems to imply the need for a rather motionless process.

One important consequence is that inhibition assays based on different sHz production protocols do not necessarily give the same drug inhibitory concentrations [24–27]. It can be argued that it may not be essential if an in vitro assay does not use the true in vivo components and mechanism, if the obtained in vitro results are applicable to what is observed in vivo. As an analogy, in laboratory medicine different “non-physiological reactions” are used, for example in enzymology, coagulation or microbiology, which are highly useful to predict in vivo effects [62].

The HLC growth is inhibited in the presence of anti-malarial compounds which also inhibit the formation of β-haematin in diverse assays [10, 15–17, 63]; and results read at 48 h are in agreement with published results [24–27] (Additional file 1).

One proposed mechanism of quinolone drugs is that the compounds adsorb onto the fastest growing face of Hz crystals in order to impede their further growth [64, 65]. However, alternatively it is thought that the drugs may form complexes with free Fe(III)PPIX in solution either through π-stacking or coordination [66]. In Raman spectrum analysis of the HLCs in the presence of CQ (Fig. 6), the Raman lines are at 1086 and 1556 cm^−1^ in CQ red-shift. The same is observed for the Raman spectra for CQ incubated with haemin, sHz or nHz (Additional file 6). These lines correspond to in-phase stretching vibrations of the quinoline ring. Such shifts seem to indicate that the interaction between the haemin molecules in HLC and CQ occurs mainly via a pyrrole-quinoline ring π–π interaction, as previously described for the interaction between CQ and β-haematin [63]. Thus the HLC assay, despite its structural difference, may predict Hz inhibiting drug effects reasonably well (Table 2). The HLC assay can be read after 24 h, although the inhibition might appear slightly more pronounced for some compounds, maximally by one concentration step (Table 2). The assay is robust and shows a good reproducibility (see Additional file 7).

Interestingly, artemisinins show a rather strong inhibitory effect (Table 2). The mechanism of action of artemisinins (ARTs) is still debated [17, 18], but one hypothesis is that ARTs may be activated by the reduced iron (Fe^2+^) or haem [17]. However, although at least four hypothesis exist on the mode of action (MOA) of ARTs [18], it remains unclear which one is correct, including doubts if the observed inhibition is appropriately measured [28]. Tetracyclines also show a weak inhibitory effect (only at 500 or 1000 µM) on HLCs growth (Table 2). Current evidence points to the apicoplast as target [67], which means that inhibiting effects are only seen in the second generation in in vitro parasite growth assays [68]. However, a weak inhibitory effect has been observed during the first cycle and no obvious explanation for this MOA was found [68].

Perhaps, more relevant than the possible mechanisms and their interpretation is the fact that the HLC assay was able to detect the inhibitory effects of artemisinins and tetracyclines. One can certainly argue that these are “false positives” with regards to drugs, like the quinolones, with a MOA of inhibiting Hz formation. However, it underlines the potential usefulness of the HLC assay in not missing any possible Hz inhibiting compounds (“false negatives”). In this context and as reported before [37], this assay may have further applications like the rapid and simplistic screening of prion inhibiting compounds.

In summary, this study investigated and optimized extensively the conditions of the HLC assay and provides data on its usefulness as an alternative method to determine inhibiting properties of anti-malarial compounds, even if the end-product is not structurally identical to Hz. Moreover, the HLC assay is simple, inexpensive, robust, reproducible, and does not require multiple manipulation steps or complex measurements of the end product. Reliable HLCs growth requires *Mycoplasma* Broth Base, Tween 80, any type of lysed blood or haemin and pancreatin, and the assay can be performed under basic laboratory conditions with a simple visual positive/negative read-out. Although HLCs are not identical to sHz, the results of the drugs inhibitory effects, especially of the quinolone and arylamino alcohol drugs, including their different potency, are consistent with those reported in the literature.

## Additional files

10.1186/s12936-015-0913- Comparison of 50 % inhibitory concentration (IC_50_) from different haemozoin inhibition assays.
10.1186/s12936-015-0913- Results from different Medium conditions after 5-7 days of incubation.
10.1186/s12936-015-0913- HLC growth over incubation period in the presence of chloroquine.
10.1186/s12936-015-0913- Supporting information - Raman analysis of haemozoin-like crystals, natural and synthetic Hz and interactions with chloroquine.
10.1186/s12936-015-0913- Observed Raman lines (cm^−1^), assignments, symmetry terms and local coordinates, for Haemin (Fe(III)-protoporphyrin IX), crude native Haemozoin, synthesized β–Haematin and Haemozoin-like crystals (HLC).
10.1186/s12936-015-0913- Raman spectrums of haemin, haemozoin and β-haematin interaction with chloroquine.
10.1186/s12936-015-0913- Reproducibility of the HLC inhibition assay.
