# Supplementary material for: Characterization and optimization of the haemozoin-like crystal (HLC) assay to determine Hz inhibiting effects of anti-malarial compounds
Source: Malar J. 2015 Oct 12;14:403. doi: 10.1186/s12936-015-0913-y (PMC4603294; doi:10.1186/s12936-015-0913-y)
Supplement: Supplementary file 1 — 10.1186/s12936-015-0913- Comparison of 50 % inhibitory concentration (IC50) from different haemozoin inhibition assays. [file 12936_2015_913_MOESM1_ESM.docx]

**Additional file 1**

**Characterization and optimization of the haemozoin-like crystal (HLC) assay to determine Hz inhibiting effects of anti-malarial compounds**

Authors: Carolina Tempera^1^, Ricardo Franco^2^, Carlos Caro^2^, Vânia André^3^, Peter Eaton^4^, Peter Burke^5^, Thomas Hänscheid^1,6^

Corresponding author E.mail: [t.hanscheid@fm.ul.pt](mailto:t.hanscheid@fm.ul.pt)

**Affiliations:**

^1^ Instituto de Medicina Molecular, Faculdade de Medicina de Lisboa, Av. Prof. Egas Moniz, P-1649-028 Lisbon, Portugal, Tel: +351 217999458, Fax: +351 217999459

^2^ UCIBIO, REQUIMTE, Departamento de Química, Faculdade de Ciências e Tecnologia, Universidade NOVA de Lisboa, 2829-516 Caparica, Portugal

^3^ Centro de Química Estrutural, Instituto Superior Técnico, Universidade de Lisboa, Av. Rovisco Pais, 1049-001 Lisbon, Portugal.

^4^ REQUIMTE/UCIBIO, Departamento de Química e Bioquímica, Faculdade de Ciências, Universidade do Porto, 4169-007 Porto, Portugal

^5^ STERIS Corporation - 5960 Heisley Road - Mentor, OH 44060, USA

^6^ Instituto de Microbiologia, Faculdade de Medicina, Lisbon, Portugal

This file includes: Comparison table of 50% inhibitory concentration (IC_50_) results from different haemozoin inhibition assays

# **Additional Table 1- Comparison of 50% inhibitory concentration (IC_50_) from different haemozoin inhibition assays**

| IC50 | HPA (µM) [[1](#_ENREF_1)] | HPA (µM) [[2](#_ENREF_2)] | | (µM) [[3](#_ENREF_3)] | CFHCS assay (µM) [[4](#_ENREF_4)] | BHIA  (equiv.) ^a^ [[5](#_ENREF_5)] | Phib  (equiv.) ^a^ [[6](#_ENREF_6)] | HLC  assay ^b^  (µM) |
| --- | --- | --- | --- | --- | --- | --- | --- | --- |
|  |  | pH 4.8 | pH 6.5 |  |  |  |  |  |
| Chloroquine | 24.4 | 45 | 11 | 252 | 374 | 1.6 | 1.9 | 62.5 |
| Amodiaquine | 15.1 | 60 | 60 | 167 | 67 | 0.8 | 1.4 | 62.5 |
| Mefloquine | 46.9 | 120 | 60 | 315 | 476 | 6.2 | 2.9 | 250 |
| Quinine | 64.8 | 160 | 25 | 365 |  | 5.5 | 3.5 | 125 |
| Halofantrine | 184.5 | 30 | 50 |  |  |  | 0.2 | 250 |
| Artemisinin |  |  |  |  |  |  |  | 250 |
| Gentamycin |  |  |  |  |  |  |  | >1000 |

a: IC_50_ values in the Phiβ assay were reported in molar equivalents of drug to heme.

b: Compound concentration presenting no visual growth in the medium with hemin 5 mM and 2.5% Pancreatin (see Additional file 4)

1. Hawley SR, Bray PG, Mungthin M, Atkinson JD, O'Neill PM, Ward SA: **Relationship between antimalarial drug activity, accumulation, and inhibition of heme polymerization in Plasmodium falciparum in vitro.** *Antimicrob Agents Chemother* 1998, **42:**682-686.

2. Dorn A, Vippagunta SR, Matile H, Jaquet C, Vennerstrom JL, Ridley RG: **An assessment of drug-haematin binding as a mechanism for inhibition of haematin polymerisation by quinoline antimalarials.** *Biochem Pharmacol* 1998, **55:**727-736.

3. Huy NT, Uyen DT, Maeda A, Trang DT, Oida T, et al: **Simple colorimetric inhibition assay of heme crystallization for high-throughput screening of antimalarial compounds.** *Antimicrob Agents Chemother* 2007, **51:**350-353.

4. Rush MA, Baniecki ML, Mazitschek R, Cortese JF, Wiegand R, et al: **Colorimetric high-throughput screen for detection of heme crystallization inhibitors.** *Antimicrob Agents Chemother* 2009, **53:**2564-2568.

5. Parapini S, Basilico N, Pasini E, Egan TJ, Olliaro P, et al: **Standardization of the physicochemical parameters to assess in vitro the beta-hematin inhibitory activity of antimalarial drugs.** *Exp Parasitol* 2000, **96:**249-256.

6. Ncokazi KK, Egan TJ: **A colorimetric high-throughput beta-hematin inhibition screening assay for use in the search for antimalarial compounds.** *Anal Biochem* 2005, **338:**306-319.
