# Supplementary material for: Characterization and optimization of the haemozoin-like crystal (HLC) assay to determine Hz inhibiting effects of anti-malarial compounds
Source: Malar J. 2015 Oct 12;14:403. doi: 10.1186/s12936-015-0913-y (PMC4603294; doi:10.1186/s12936-015-0913-y)
Supplement: Supplementary file 2 — 10.1186/s12936-015-0913- Results from different Medium conditions after 5-7 days of incubation. [file 12936_2015_913_MOESM2_ESM.docx]

**Additional file 2**

**Characterization and optimization of the haemozoin-like crystal (HLC) assay to determine Hz inhibiting effects of anti-malarial compounds**

Authors: Carolina Tempera^1^, Ricardo Franco^2^, Carlos Caro^2^, Vânia André^3^, Peter Eaton^4^, Peter Burke^5^, Thomas Hänscheid^1,6^

Corresponding author E.mail: [t.hanscheid@fm.ul.pt](mailto:t.hanscheid@fm.ul.pt)

**Affiliations:**

^1^ Instituto de Medicina Molecular, Faculdade de Medicina de Lisboa, Av. Prof. Egas Moniz, P-1649-028 Lisbon, Portugal, Tel: +351 217999458, Fax: +351 217999459

^2^ UCIBIO, REQUIMTE, Departamento de Química, Faculdade de Ciências e Tecnologia, Universidade NOVA de Lisboa, 2829-516 Caparica, Portugal

^3^ Centro de Química Estrutural, Instituto Superior Técnico, Universidade de Lisboa, Av. Rovisco Pais, 1049-001 Lisbon, Portugal.

^4^ REQUIMTE/UCIBIO, Departamento de Química e Bioquímica, Faculdade de Ciências, Universidade do Porto, 4169-007 Porto, Portugal

^5^ STERIS Corporation - 5960 Heisley Road - Mentor, OH 44060, USA

^6^ Instituto de Microbiologia, Faculdade de Medicina, Lisbon, Portugal

This file includes: Result table from different tested medium conditions

**Additional Table 2 – Results from different Medium conditions after 5-7 days of incubation**

| Condition | | Result | | | | | | | | | | |
| --- | --- | --- | --- | --- | --- | --- | --- | --- | --- | --- | --- | --- |
| Medium pH5 ^a^ | | false positive | | | | | | | | | | |
| Medium pH6 ^a^ | | false positive | | | | | | | | | | |
| Medium pH8 ^b^ | | yes | | | | | | | | | | |
| Medium pH9 ^b^ | | false positive | | | | | | | | | | |
| Medium pH11 ^b^ | | no | | | | | | | | | | |
| IFDO seeding concentration: 0.01µM | | yes (slightly longer period than the control) | | | | | | | | | | |
| IFDO seeding concentration: 0.5 µM | | yes (slightly quicker than the control) | | | | | | | | | | |
| sHz seeding concentration: 0.01µM | | yes (takes more time than with IFDO seeding) | | | | | | | | | | |
| sHz seeding concentration: 0.1µM | | yes (takes more time than with IFDO seeding) | | | | | | | | | | |
| sHz seeding concentration: 0.5 µM | | yes (takes more time than with IFDO seeding) | | | | | | | | | | |
| Medium without serum | | | yes | | | | | | | | |  |
| Without Tween 80 | | | no | | | | | | | | |  |
| Tween 80 at 1 mL/L | | | yes | | | | | | | | |  |
| Tween 80 at 4 mL/L | | | yes | | | | | | | | |  |
| Tween 80 at 8 mL/L | | | yes | | | | | | | | |  |
| Medium without lysed blood | | | no | | | | | | | | |  |
| Sheep blood extract | | | yes | | | | | | | | |  |
| Lysed blood at 15 mL/L | | | yes | | | | | | | | |  |
| Hemin in NaOH (10 mM) | | | false positive | | | | | | | | |  |
| Medium without Pancreatin | | | no | | | | | | | | |  |
| Pancreatin 10% at 10 mL/L | | | yes | | | | | | | | |  |
| Pancreatin 2.5% instead of 10% | | | yes | | | | | | | | |  |
|  | | | 10% Pancreatin | | | 2.5% Pancreatin | | | | No Pancreatin | |  |
| Hemin in DMSO (10 mM) | | | yes | | | yes | | | | n.d. | |  |
| Hemin in DMSO (5m M) | | | yes | | | yes | | | | no | |  |
| Hemin in DMSO (2.5 mM) | | | yes | | | yes | | | | no | |  |
| Hemin in Tween 80 | | | Note: hemin did not dissolve | | | | | | | | |  |
| Lysed blood from different blood donors | | | yes (similar between the different lysed bloods) | | | | | | | | |  |
|  | | | | | Serum | | | Mix serum | Plasma | | No serum, no plasma |  |
| Anti-coagulated lysed blood from different donors ^c^ | | | | | yes | | | no | no | | yes |  |
| Coagulated lysed blood from different donors | | | | | no | | | n.d | no | | n.d. |  |
| Mycoplasma broth at 25.5 g/L | | | false positive | | | | | | | | |  |
| Mycoplasma broth at 15 g/L | | | false positive | | | | | | | | |  |
| Medium without mycoplasma broth  (not autoclaved Tween 80; final medium filtered whit 0.22µm filter) | | | With serum | | | | Without serum | | | | |  |
|  |  |  | no | | | | no | | | | |  |
| Medium without mycoplasma broth  (autoclaved Tween 80) | 10% Pancreatin with serum | | | 10% Pancreatin without serum | | | 2.5% Pancreatin  with serum | | | 2.5% Pancreatin  without serum | |  |
|  | false positive | | | false positive | | | false positive | | | false positive | |  |

(Cont.)

| Condition | | | Result | | | | | | | |
| --- | --- | --- | --- | --- | --- | --- | --- | --- | --- | --- |
| Medium without mycoplasma broth (autoclaved tween80) at 10% Pancreatin | | | | | | | | | | |
| Hemin 10mM, with serum | | false positive | | | | | | | | |
| Hemin 10 mM without serum | | false positive | | | | | | | | |
| Hemin 5mM, with serum | | false positive | | | | | | | | |
| Hemin 5mM, without serum | | no | | | | | | | | |
| Hemin 2.5mM, with serum | | no | | | | | | | | |
| Hemin 2.5mM, without serum | | no | | | | | | | | |
| Incubation at 50ºC  (Medium not autoclaved nor filtered – without mycoplasma broth) | | | | With tween | | | | | Without tween | |
| Pancreatin at 10% | | | | false positive | | | | | no | |
| Pancreatin at 8% | | | | false positive | | | | | no | |
| Pancreatin at 6% | | | | false positive | | | | | no | |
| Pancreatin at 4% | | | | no | | | | | no | |
| Pancreatin at 2% | | | | no | | | | | no | |
| Incubation at 37ºC, ambient atmosphere | yes | | | | | | | | | |
| Incubation at 35ºC, ambient atmosphere | false positive | | | | | | | | | |
|  | Blood 10% | | | | | Blood 2.5% | | Hemin 10% | | Hemin 2.5% |
| Incubation at 37ºC  (5%O_2_ ; 5%CO_2_; 90%N2) | yes | | | | | yes | | yes | | yes |
| Room temperature and ambient atmosphere | false positive | | | | | false positive | | false positive | | false positive |
| Using PBS10x instead of water | | | | no | | | | | | |
|  | | | | | IFDO seeding | | sHz seeding | | | |
| Using PBS at 1x instead of water | | | | | yes | | false positive | | | |
| Using PBS at 2x instead of water | | | | | yes | | false positive | | | |
| Using PBS at 3x instead of water | | | | | no | | no | | | |

Baseline conditions as for the IFDO growing medium (see methods): *Mycoplasma* broth (35.5 g/L), Tween 80 (2 mL/L), Lysed Blood (30 mL/L), Pancreatin 10% (20 mL/L) and Horse Serum (1.33 mL/L). Results observed during the 5-7 days of incubation.

Yes - means that positive growth was visible in the end of the incubation period. The growth presented at the same time as the baseline medium or took longer, not suggesting an improvement to the baseline medium.

False positive - means the some black growth is present however not with the same aspect as the baseline medium. Which mean that the medium presents darker or the growth occurs at the same time in seeded and not seeded medium.

No - refers to conditions where black sediment growth never was observed during the 7 days of incubation

n.d. – not done

*a* pH changed with sulfuric acid

*b* pH changed with sodium hydroxide

*c* Heparin was used for anti-coagulated blood
