# Supplementary material for: Characterization and optimization of the haemozoin-like crystal (HLC) assay to determine Hz inhibiting effects of anti-malarial compounds
Source: Malar J. 2015 Oct 12;14:403. doi: 10.1186/s12936-015-0913-y (PMC4603294; doi:10.1186/s12936-015-0913-y)
Supplement: Supplementary file 3 — 10.1186/s12936-015-0913- HLC growth over incubation period in the presence of chloroquine. [file 12936_2015_913_MOESM3_ESM.docx]

**Additional file 3**

**Characterization and optimization of the haemozoin-like crystal (HLC) assay to determine Hz inhibiting effects of anti-malarial compounds**

Authors: Carolina Tempera^1^, Ricardo Franco^2^, Carlos Caro^2^, Vânia André^3^, Peter Eaton^4^, Peter Burke^5^, Thomas Hänscheid^1,6^

Corresponding author E.mail: [t.hanscheid@fm.ul.pt](mailto:t.hanscheid@fm.ul.pt)

**Affiliations:**

^1^ Instituto de Medicina Molecular, Faculdade de Medicina de Lisboa, Av. Prof. Egas Moniz, P-1649-028 Lisbon, Portugal, Tel: +351 217999458, Fax: +351 217999459

^2^ UCIBIO, REQUIMTE, Departamento de Química, Faculdade de Ciências e Tecnologia, Universidade NOVA de Lisboa, 2829-516 Caparica, Portugal

^3^ Centro de Química Estrutural, Instituto Superior Técnico, Universidade de Lisboa, Av. Rovisco Pais, 1049-001 Lisbon, Portugal.

^4^ REQUIMTE/UCIBIO, Departamento de Química e Bioquímica, Faculdade de Ciências, Universidade do Porto, 4169-007 Porto, Portugal

^5^ STERIS Corporation - 5960 Heisley Road - Mentor, OH 44060, USA

^6^ Instituto de Microbiologia, Faculdade de Medicina, Lisbon, Portugal

This file includes: Representative growth of HLCs over incubation period as well as in presence of chloroquine


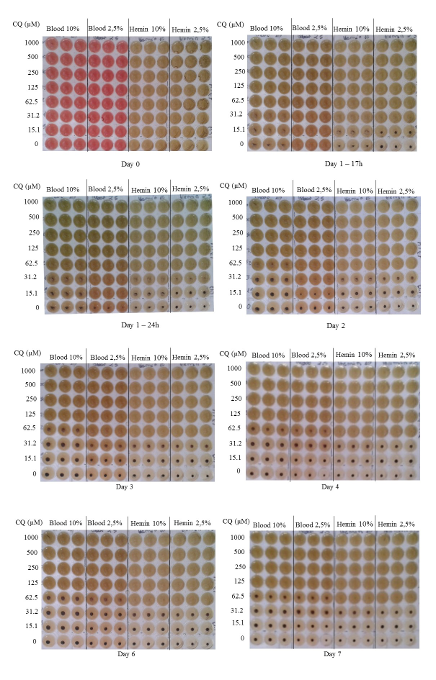


**Figure 1 - HLCs growth over incubation period in the presence of chloroquine.** Representative daily pictures of a 96 well-plate with the four tested medium for HLCs growth. After 24 hours or less, the mediums, with the exception of the medium with blood and 2.5% Pancreatin, show visual growth (the dark dot) without the presence of any compound. The presence of growth also reflects a change in medium color. In terms of inhibition, the medium with hemin instead of blood presents a final result by day 2. However, the medium with blood present growth in a higher compound concentration if in a longer period of incubation. For a better perception of growth presence a spin down was done, by plate centrifugation.
