# Supplementary material for: Characterization and optimization of the haemozoin-like crystal (HLC) assay to determine Hz inhibiting effects of anti-malarial compounds
Source: Malar J. 2015 Oct 12;14:403. doi: 10.1186/s12936-015-0913-y (PMC4603294; doi:10.1186/s12936-015-0913-y)
Supplement: Supplementary file 4 — 10.1186/s12936-015-0913- Supporting information - Raman analysis of haemozoin-like crystals, natural and synthetic Hz and interactions with chloroquine. [file 12936_2015_913_MOESM4_ESM.docx]

**Additional file 4**

**Characterization and optimization of the haemozoin-like crystal (HLC) assay to determine Hz inhibiting effects of anti-malarial compounds**

Authors: Carolina Tempera^1^, Ricardo Franco^2^, Carlos Caro^2^, Vânia André^3^, Peter Eaton^4^, Peter Burke^5^, Thomas Hänscheid^1,6^

Corresponding author E.mail: [t.hanscheid@fm.ul.pt](mailto:t.hanscheid@fm.ul.pt)

**Affiliations:**

^1^ Instituto de Medicina Molecular, Faculdade de Medicina de Lisboa, Av. Prof. Egas Moniz, P-1649-028 Lisbon, Portugal, Tel: +351 217999458, Fax: +351 217999459

^2^ UCIBIO, REQUIMTE, Departamento de Química, Faculdade de Ciências e Tecnologia, Universidade NOVA de Lisboa, 2829-516 Caparica, Portugal

^3^ Centro de Química Estrutural, Instituto Superior Técnico, Universidade de Lisboa, Av. Rovisco Pais, 1049-001 Lisbon, Portugal.

^4^ REQUIMTE/UCIBIO, Departamento de Química e Bioquímica, Faculdade de Ciências, Universidade do Porto, 4169-007 Porto, Portugal

^5^ STERIS Corporation - 5960 Heisley Road - Mentor, OH 44060, USA

^6^ Instituto de Microbiologia, Faculdade de Medicina, Lisbon, Portugal

This file includes: Supporting information of Raman analysis of hemozoin-like crystals, natural and sythethic Hz and interactions with choloroquine.

**Raman analysis of hemozoin-like crystals, natural and sythethic Hz and interactions with choloroquine.**

Ricardo Franco; Email: [ricardo.franco@fct.unl.pt](mailto:ricardo.franco@fct.unl.pt) and Carlos Caro; Email: cacarsal@gmail.com

Comparing the Raman spectrum of HLCs with the other hemin-containing species, it becomes obvious that HLCs present a Raman spectrum that is more similar to free hemin than to nHz or sHz. In other words, the hemin present in HLCs probably presents a structure that is more similar to the π-π aggregated structure observed for hemin in aqueous solution, in opposition to the crystalline structures present in nHz and sHz [1]. These lines correspond to in phase stretching vibrations of the quinoline ring [2]. At low frequency, HLC Raman lines related to out-of-plane stretches, red-shift when in the presence of CQ (Figure 6, compare spectra A and B). These are the line at 971 cm^-1^ (assigned to a ν_46_ mode, a pyrrole asymmetric deformation mode – Additional file 5) that red-shifts to 966 cm^-1^; and the line corresponding to an out-of-plane γ_10_ vibration, that is blue-shifted to 839 cm^-1^ in HLC in relation to the Raman spectrum of nHz and sHz (Figure 5), and that in the HLC/CQ mixture red-shifts back to its original position at 820 cm^-1^. As these low-frequency lines are related to heme pyrrole-ring deformations, such results seem to indicate that the interaction between HLC and CQ eliminates some of the out-of-plane distortion of the hemin present in HLC. Analysis was based on previous studies on metmyoglobin [3] and β–hematin [4], and are presented in Table S1. Namely, the HLCs Raman spectrum presents a vinyl =CH_2_ scissor mode, a deformation mode occurring at 1460 cm^-1^ in HLC (Fig. 5D). This mode is strongly coupled with the symmetric ν_28_ mode (1430 cm^-1^) [3], a symmetric in-plane stretch. Interestingly, the ν_28_ mode has a very prominent intensity relative to the vinyl =CH_2_ scissor mode in the case of hemin, nHZ and sHz. Conversely, the intensity of the deformation vinyl =CH_2_ scissor in the HLC spectrum is extremely high relative to an almost inexistent symmetric ν_28_ mode. Another line that is prominent in the HLC spectrum relative to the spectra of the other heme-containing species, corresponds to an out-of-plane vibration mode occurring at 971 cm^-1^ and assigned to ν_46_, a pyrrole asymmetric deformation mode [4]. As further evidence for hemin in HLC being more distorted than in the other hemin-containing counterparts, the line appearing at around 820 cm^-1^ for hemin, nHz and sHz, corresponds to out-of-plane γ_10_ vibration modes, and presents an extreme ca. 20 cm^-1^ blue-shift to 839 cm^-1^ in HLC.

1. Solomonov I, Osipova M, Feldman Y, Baehtz C, Kjaer K, Robinson IK, et al: **Crystal nucleation, growth, and morphology of the synthetic malaria pigment beta-hematin and the effect thereon by quinoline additives: the malaria pigment as a target of various antimalarial drugs.** *J Am Chem Soc* 2007, **129:**2615-2627.

2. Frosch T, Koncarevic S, Zedler L, Schmitt M, Schenzel K, et al: **In situ localization and structural analysis of the malaria pigment hemozoin.** *J Phys Chem B* 2007, **111:**11047-11056.

3. Hu S, Smith KM, Spiro TG: **Assignment of Protoheme Resonance Raman Spectrum by Heme Labeling in Myoglobin.** *Journal of the American Chemical Society* 1996, **118:**12638-12646.

4. Wood BR, Langford SJ, Cooke BM, Lim J, Glenister FK, Duriska M, et al: **Resonance Raman spectroscopy reveals new insight into the electronic structure of beta-hematin and malaria pigment.** *J Am Chem Soc* 2004, **126:**9233-9239.
