# Supplementary material for: Characterization and optimization of the haemozoin-like crystal (HLC) assay to determine Hz inhibiting effects of anti-malarial compounds
Source: Malar J. 2015 Oct 12;14:403. doi: 10.1186/s12936-015-0913-y (PMC4603294; doi:10.1186/s12936-015-0913-y)
Supplement: Supplementary file 5 — 10.1186/s12936-015-0913- Observed Raman lines (cm−1), assignments, symmetry terms and local coordinates, for Haemin (Fe(III)-protoporphyrin IX), crude native Haemozoin, synthesized β–Haematin and Haemozoin-like crystals (HLC). [file 12936_2015_913_MOESM5_ESM.docx]

**Additional file 5**

**Characterization and optimization of the haemozoin-like crystal (HLC) assay to determine Hz inhibiting effects of anti-malarial compounds**

Authors: Carolina Tempera^1^, Ricardo Franco^2^, Carlos Caro^2^, Vânia André^3^, Peter Eaton^4^, Peter Burke^5^, Thomas Hänscheid^1,6^

Corresponding author E.mail: [t.hanscheid@fm.ul.pt](mailto:t.hanscheid@fm.ul.pt)

**Affiliations:**

^1^ Instituto de Medicina Molecular, Faculdade de Medicina de Lisboa, Av. Prof. Egas Moniz, P-1649-028 Lisbon, Portugal, Tel: +351 217999458, Fax: +351 217999459

^2^ UCIBIO, REQUIMTE, Departamento de Química, Faculdade de Ciências e Tecnologia, Universidade NOVA de Lisboa, 2829-516 Caparica, Portugal

^3^ Centro de Química Estrutural, Instituto Superior Técnico, Universidade de Lisboa, Av. Rovisco Pais, 1049-001 Lisbon, Portugal.

^4^ REQUIMTE/UCIBIO, Departamento de Química e Bioquímica, Faculdade de Ciências, Universidade do Porto, 4169-007 Porto, Portugal

^5^ STERIS Corporation - 5960 Heisley Road - Mentor, OH 44060, USA

^6^ Instituto de Microbiologia, Faculdade de Medicina, Lisbon, Portugal

This file includes: Table with observed Raman lines (cm^−1^), assignments, symmetry terms and local coordinates, for Hemin (Fe(III)-protoporphyrin IX), crude native Hemozoin, synthesized β–Hematin and Hemozoin-Like Crystals (HLC).

**Additional Table 3** - Observed Raman lines (cm^−1^), assignments, symmetry terms and local coordinates, for Hemin (Fe(III)-protoporphyrin IX), crude native Hemozoin, synthesized β–Hematin and Hemozoin-Like Crystals (HLC).

| **Hemin**  **(cm^-1^)** | **Hemozoin (cm^-1^)** | **β-hematin (cm^-1^)** | **HLC (cm^-1^)** | **Assignment** | **Symmetry terms** | **Local coordinate** |
| --- | --- | --- | --- | --- | --- | --- |
| 1621 | 1625 | 1626 | 1626 | ν _10_ | B_1g_ | ν (C_α_C_m_)_asym_ |
| 1551 | 1551 | 1548 | 1551 | ν _11_ | B_1g_ | ν (C_β_C_β_) |
| 1566 | 1568 | 1569 | 1570 | ν _2_ | A_1g_ | ν (C_β_C_β_) |
| 1488 | 1487 | 1493 | 1490 | ν _3_ | A_1g_ | ν (C_α_C_m_)_sym_ |
| absent | absent | absent | 1460 |  |  | δ(=C_b_H_2_)_s_ |
| 1430 | 1429 | 1429 | absent | ν _28_ | B_2g_ | ν (C_α_C_m_)_sym_ |
| 1395 | 1399 | 1399 | 1392 | ν _20_ or ν _29_ | A_2g_ or B_2g_ | ν (pyr quarter-ring) |
| 1371 | 1371 | 1371 | 1365 | ν _4_ | A_1g_ | ν (pyr half-ring)_sym_ |
| 1339 | 1339 | 1340 | 1330 | ν _41_ | E_u_ | ν (pyr half-ring)_sym_ |
| 1307 | 1307 | 1307 | 1310 | ν _21_ | A_2g_ | δ(C_m_H) |
| 1234 | 1242 | 1242 | 1242 | ν _42_ | E_u_ | δ(C_m_H) |
| 1220 | 1218 | 1221 | 1218 | ν _13_ | B_1g_ | δ(C_m_H) |
| 1168 | 1170 | 1169 | 1163 | ν _30_ | B_2g_ | ν (pyr half-ring)_asym_ |
| absent | 1146 | 1146 | 1146 | ν _14_ | B_1g_ | ν (C_β_C_1_)_sym_ |
| 1126 | 1121 | 1120 | 1128 | ν _5_ | A_1g_ | ν (C_β_-methyl) |
| 1086 | 1085 | 1093 | 1086 |  |  | δ (=C_b_H_2_)_asym_ |
| absent | 1075 | 1079 | absent |  |  | δ (=C_b_H_2_)_asym_ |
| 1000 | 1001 | 1006 | 1000 | ν _45_ | E_u_ | ν (C_β_-vinyl) |
| 971 | 971 | 973 | 971 | ν _46 *_ | E_u_ | δ(pyr deform)_asym_ |
| 819 | 820 | 821 | 839 | γ _10_ | B_1u_ | γ (C_m_H) |
| 798 | 798 | 795 | 807 | ν _6_ | A_1g_ | ν (pyr breathing) |
| 753 | 754 | 754 | 754 | ν _15_ | B_1g_ | ν (pyr breathing) |

*- Wood et al. [1] assign the line around 970 cm^-1^ to ν_46_. Nevertheless, Hu et al.[2]assign a spectral feature at 989 cm^-1^ to the out-of-plane C_a_H= wag, and mention that this band seems to be derived only from the 2-vinyl group. Hu et al. assign ν_46_ to another line, appearing at 930 cm^-1^.

Vinyl related modes are in bold. ν = in-plane stretch, γ = out-of-plane stretch, δ = deformation mode.

1. Wood BR, Langford SJ, Cooke BM, Lim J, Glenister FK, Duriska M, Unthank JK, McNaughton D: **Resonance Raman Spectroscopy Reveals New Insight into the Electronic Structure of β-Hematin and Malaria Pigment.** *Journal of the American Chemical Society* 2004, **126:**9233-9239.

2. Hu S, Smith KM, Spiro TG: **Assignment of Protoheme Resonance Raman Spectrum by Heme Labeling in Myoglobin.** *Journal of the American Chemical Society* 1996, **118:**12638-12646.
