# Supplementary material for: Characterization and optimization of the haemozoin-like crystal (HLC) assay to determine Hz inhibiting effects of anti-malarial compounds
Source: Malar J. 2015 Oct 12;14:403. doi: 10.1186/s12936-015-0913-y (PMC4603294; doi:10.1186/s12936-015-0913-y)
Supplement: Supplementary file 6 — 10.1186/s12936-015-0913- Raman spectrums of haemin, haemozoin and β-haematin interaction with chloroquine. [file 12936_2015_913_MOESM6_ESM.docx]

**Additional file 6**

**Characterization and optimization of the haemozoin-like crystal (HLC) assay to determine Hz inhibiting effects of anti-malarial compounds**

Authors: Carolina Tempera^1^, Ricardo Franco^2^, Carlos Caro^2^, Vânia André^3^, Peter Eaton^4^, Peter Burke^5^, Thomas Hänscheid^1,6^

Corresponding author E.mail: [t.hanscheid@fm.ul.pt](mailto:t.hanscheid@fm.ul.pt)

**Affiliations:**

^1^ Instituto de Medicina Molecular, Faculdade de Medicina de Lisboa, Av. Prof. Egas Moniz, P-1649-028 Lisbon, Portugal, Tel: +351 217999458, Fax: +351 217999459

^2^ UCIBIO, REQUIMTE, Departamento de Química, Faculdade de Ciências e Tecnologia, Universidade NOVA de Lisboa, 2829-516 Caparica, Portugal

^3^ Centro de Química Estrutural, Instituto Superior Técnico, Universidade de Lisboa, Av. Rovisco Pais, 1049-001 Lisbon, Portugal.

^4^ REQUIMTE/UCIBIO, Departamento de Química e Bioquímica, Faculdade de Ciências, Universidade do Porto, 4169-007 Porto, Portugal

^5^ STERIS Corporation - 5960 Heisley Road - Mentor, OH 44060, USA

^6^ Instituto de Microbiologia, Faculdade de Medicina, Lisbon, Portugal

This file includes: Raman spectrums of hemin, hemozoin and β-hematin when incubated with chloroquine


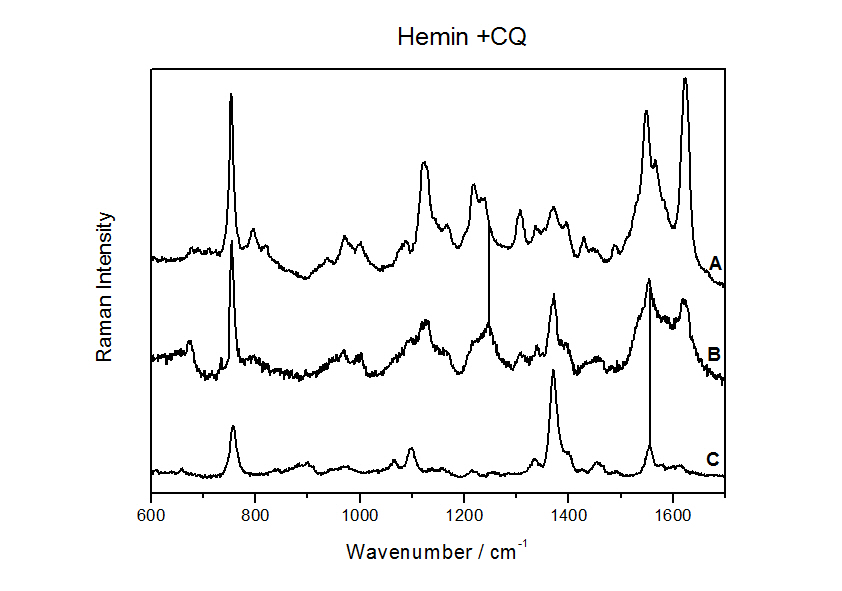


**Figure 1** - Comparison of Raman spectra of hemin (A), hemin incubated with chloroquine (B), and chloroquine alone (C). Spectra were obtained using 632.8 nm laser excitation. Vertical lines indicate Raman line shifts.


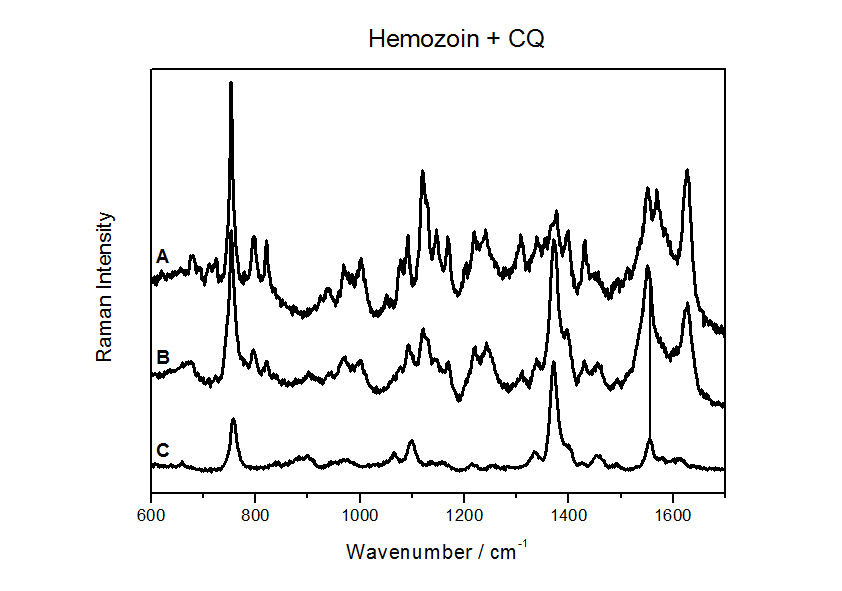


**Figure 2** - Comparison of Raman spectra of haemozoin (A), haemozoin incubated with chloroquine (B), and chloroquine alone (C). Spectra were obtained using 632.8 nm laser excitation. Vertical lines indicate Raman line shifts.


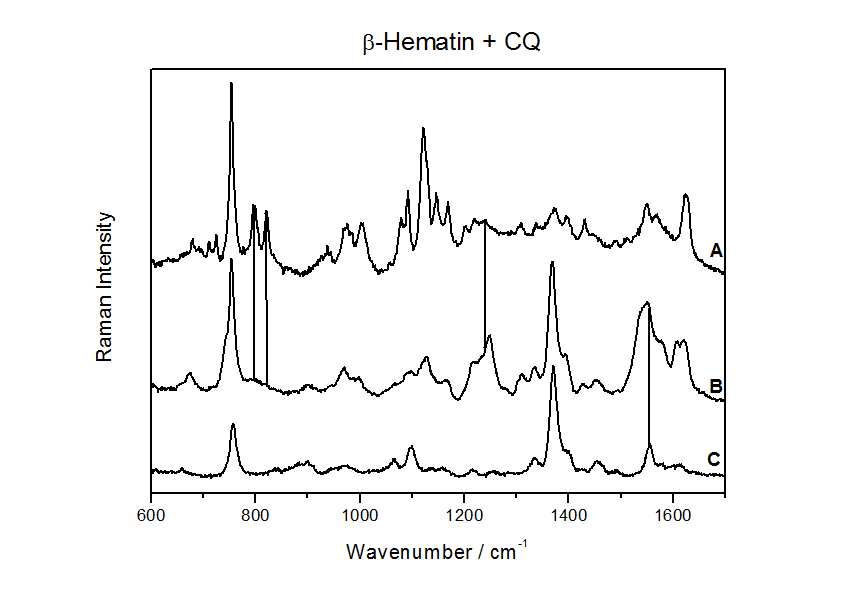


**Figure 3** - Comparison of Raman spectra of β-hematin (A), β-hematin incubated with chloroquine (B), and chloroquine alone (C). Spectra were obtained using 632.8 nm laser excitation. Vertical lines indicate Raman line shifts.
