# Supplementary material for: Characterization and optimization of the haemozoin-like crystal (HLC) assay to determine Hz inhibiting effects of anti-malarial compounds
Source: Malar J. 2015 Oct 12;14:403. doi: 10.1186/s12936-015-0913-y (PMC4603294; doi:10.1186/s12936-015-0913-y)
Supplement: Supplementary file 7 — 10.1186/s12936-015-0913- Reproducibility of the HLC inhibition assay. [file 12936_2015_913_MOESM7_ESM.docx]

**Additional file 7**

**Characterization and optimization of the haemozoin-like crystal (HLC) assay to determine Hz inhibiting effects of anti-malarial compounds**

Authors: Carolina Tempera^1^, Ricardo Franco^2^, Carlos Caro^2^, Vânia André^3^, Peter Eaton^4^, Peter Burke^5^, Thomas Hänscheid^1,6^

Corresponding author E.mail: [t.hanscheid@fm.ul.pt](mailto:t.hanscheid@fm.ul.pt)

**Affiliations:**

^1^ Instituto de Medicina Molecular, Faculdade de Medicina de Lisboa, Av. Prof. Egas Moniz, P-1649-028 Lisbon, Portugal, Tel: +351 217999458, Fax: +351 217999459

^2^ UCIBIO, REQUIMTE, Departamento de Química, Faculdade de Ciências e Tecnologia, Universidade NOVA de Lisboa, 2829-516 Caparica, Portugal

^3^ Centro de Química Estrutural, Instituto Superior Técnico, Universidade de Lisboa, Av. Rovisco Pais, 1049-001 Lisbon, Portugal.

^4^ REQUIMTE/UCIBIO, Departamento de Química e Bioquímica, Faculdade de Ciências, Universidade do Porto, 4169-007 Porto, Portugal

^5^ STERIS Corporation - 5960 Heisley Road - Mentor, OH 44060, USA

^6^ Instituto de Microbiologia, Faculdade de Medicina, Lisbon, Portugal

This file includes: Table presentation of HLC inhibition assay reproducibility

# **Additional Table 4- Reproducibility of the HLC inhibition assay**

| Chloroquine inhibition (µM) | Condition | | | | | | | |
| --- | --- | --- | --- | --- | --- | --- | --- | --- |
| Experiment | Lysed Blood | | | | Hemin 5 mM | | | |
|  | Pancreatin 10% | | Pancreatin 2.5% | | Pancreatin 10% | | Pancreatin 2.5% | |
|  | Day 2 | Day 7 | Day 2 | Day 7 | Day 2 | Day 7 | Day2 | Day7 |
| n.1 | 62.5 | 125 | 0 | 31.2 | 62.5 | 62.5 | 62.5 | 62.5 |
| n.2 | 125 | 125 | 62.5 | 125 | 62.5 | 62.5 | 62.5 | 62.5 |
| n.3 | 62.5 | 125 | 62.5 | 125 | 62.5 | 62.5 | 62.5 | 62.5 |
| n.4 | 125 | 125 | 31.2 | 125 | 62.5 | 62.5 | 62.5 | 62.5 |
| n.5 | 62.5 | 125 | 62.5 | 125 | 62.5 | 62.5 | 62.5 | 62.5 |
| n.6 | 62.5 | 125 | 62.5 | 125 | 62.5 | 62.5 | 62.5 | 62.5 |
| n.7 | 62.5 | 125 | 62.5 | 125 | 62.5 | 62.5 | 62.5 | 62.5 |
| n.8 | 62.5 | 125 | 0 | 125 | 62.5 | 62.5 | 62.5 | 62.5 |
| n.9 | 62.5 |  | 125 | 125 |  |  | 62.5 | 62.5 |
| n.10 |  |  | 62.5 | 125 |  |  | 62.5 | 62.5 |
| n.11 |  |  | 31.2 |  |  |  | 62.5 |  |
| n.12 |  |  |  |  |  |  | 62.5 |  |

The presented concentration correspond to the first concentration where it is not possible to observe growth (see Additional file 3). The medium with hemin gives the better reproducible result. The medium with lysed blood gives a more reproducible result in later period of incubation than the medium with hemin. Due to the fact that the medium with blood and 2.5% Pancreatin take longer to present growth it also presents a less reproducible result in fewer incubation time (by day 2)
